# The complete chloroplast genome of *Styrax faberi* Perk. (Styracaceae) 

**DOI:** 10.1080/23802359.2019.1698351

**Published:** 2019-12-12

**Authors:** Lili Tong, Xiaogang Xu, Yabo Wang, Yaoqin Zhang, Zixun Zhao

**Affiliations:** a School of Horticulture & Landscape Architecture, Jinling Institute of Technology, Nanjing, Jiangsu, China;; b College of Biology and Environment, Nanjing Forestry University, Nanjing, Jiangsu, China;; c Co-Innovation Center for Sustainable Forestry in Southern China, Nanjing Forestry University, Nanjing, Jiangsu, China

**Keywords:** *Styrax faberi*, chloroplast genome, phylogenetic tree

## Abstract

*Styrax faberi* Perk., a native plant belonging to Styracaceae mainly distributed in the region south of the Yantze River, China, is both a medicinal and landscape plant. Here we studied the complete chloroplast (cp) genome sequence of *S*. *faberi* by using next-generation sequencing. The whole complete cp genome of *S*. *faberi* totals 159,424 bp in length, containing a large single-copy (LSC) region of 85,845 bp, and a small single-copy (SSC) region of 18,027 bp 133 genes, including 8 rRNA genes, 37 tRNAs genes, and 88 protein-coding genes constituted the genome. The GC content of *S. faberi* is 36.92%. The phylogenetic analysis reveals that *S*. *faberi* is a sister species to *Styrax odoratissimus* and *Styrax zhejiangensis* in *Styracaceae*.

*Styrax faberi*, a shrub or small tree with abundant white fragrant flowers blooming in late spring, is a precious ornamental tree for landscape greening. It is suitable for embellishing courtyards or planting on hillsides (Hwang 1994). It also can be used in the medicinal field in China. However, its phylogenetic position is still a mystery, in previous studies, because of the lack of its genomic resources. In this study, we characterized the complete chloroplast (cp) genome sequence of *S. faberi* (GeneBank accession number: MN335255) based on the data of genome sequencing.

The total genomic DNA was extracted from the fresh leaves of *S. faberi* in Mountain Qiyun (29.820°N, 118.038°E), Anhui Province, China, using the DNeasy Plant Kit (Qiagen, Valencia, CA), and stored at −80 °C in the Lab of Nanjing Forestry University. The voucher specimen was reserved at Nanjing Forestry University Herbarium (accession number NF2018669). Nanjing Genepioneer Biotechnologies Inc. (Nanjing, China) was responsible for the whole genome sequencing on the platform of Illumina Hiseq. The raw reads were filtered by CLC Genomics Workbench v9, and the obtained clean reads were assembled into cp genome using SPAdes (Bankevich et al. 2012). Gene structure annotation was carried out with CpGAVAS (Liu et al. 2012) and the physical map was generated with OGDRAW (Lohse et al. 2013). A phylogenetic tree was inferred based on the maximum-likelihood (ML) method by using MAFFT (Katoh and Standley 2013).

The circular DNA of *S. faberi* totaled 159,424 bp in size and included two inverted repeat (IRa and IRb) regions of 27,776 bp each, separated by a large single copy (LSC) region of 85,845 bp, and a small single copy (SSC) region of 18,027 bp. A total of 133 genes are encoded, including 88 protein-coding genes (79 PCG species), 37 tRNA genes (27 tRNA species), and 8 rRNA genes (4 rRNA species). Among these, four PCGs (*ndhB*, *rpl2*, *rpoC1*, and *rps16*) contained one intron while the others had two introns (*clpP* and *ycf3*). The complete GC content of *S. faberi* genome is 36.92%, and the corresponding values in LSC, SSC, and IR regions are 34.84, 30.29, and 42.27%, respectively.

To investigate its taxonomic status, the phylogenetic tree was constructed by maximum-likelihood (ML) method based on the cp genome of 24 respective species from Styracaceae, Symplocaceae, Actinidaiaceae, Theaceae. The ML phylogenetic tree showed that *S. faberi* was clustered with other families of Ericales such as Symplocaceae, Actinidiaceae, Theaceae, Ericaceae, and was a sister species to *Styrax odoratissimus* and *Styrax zhejiangensis* in Styracaceae, with bootstrap support values of 100% (Figure 1).

**Figure 1.**
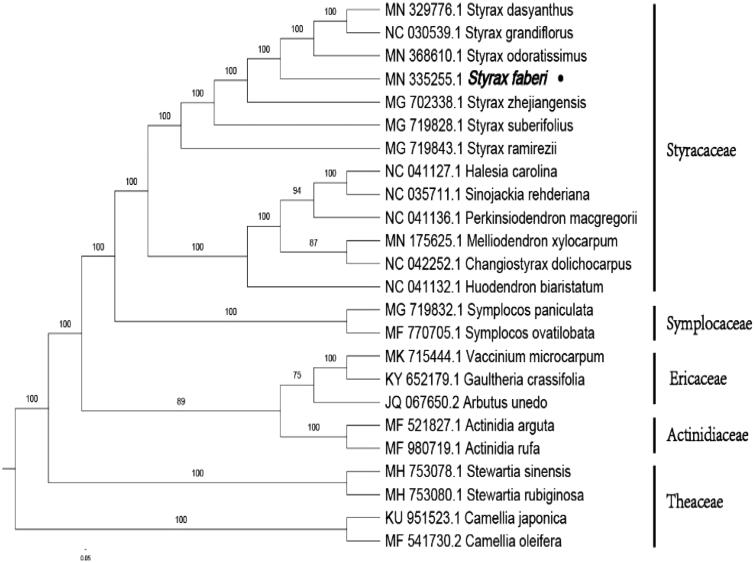
Phylogenetic tree inferred by maximum-likelihood (ML) method based on the complete chloroplast genome of 24 representative species. The bootstrap support values are shown at the branches.
